# Should breast cancer survivors be excluded from, or invited to, organised mammography screening programmes?

**DOI:** 10.1186/1472-6963-11-249

**Published:** 2011-10-04

**Authors:** Lauro Bucchi

**Affiliations:** 1 Romagna Cancer Registry, IRST, 47014 Meldola, Forlì, Italy

## Abstract

**Background:**

The prevalence of breast cancer in developed countries has steadily risen over recent decades. Immediate and long-term health needs of patients, including preventive care and screening services, are receiving increasing attention. A question still unresolved is whether breast cancer survivors should receive mammographic surveillance in the clinical or screening setting and, thus, whether they should be excluded from, or invited to, organised mammography screening programmes. The objective of this article is to discuss the many contradictory aspects of this matter.

**Discussion:**

Problems with mammographic surveillance of breast cancer survivors include: weak evidence of a reduction in mortality; lack of evidence in favour of one setting or the other; lack of evidence-based guidelines for the frequency and duration of surveillance; disproportionate emphasis placed on the first few years post-treatment, probably dictated by surgical and oncological priorities; a variety of screening policies, as these women are permanently or temporarily or partially excluded from many - but not all - organised screening programmes worldwide; an even greater disparity in follow-up protocols used in the clinical setting; a paucity of data on compliance to mammographic surveillance in both settings; and a difficulty in coordinating the roles of health care providers. In the future, the use of mammography in breast cancer survivors will be influenced by the inclusion of women aged > 69 years in organised screening programmes and the implementation of multidisciplinary breast units, and will probably be investigated by research activities on individual risk assessment and risk-tailored screening. In the interim, current problems can be partially alleviated with some technical solutions in screening data recording, patient flows, and care coordination.

**Summary:**

Mammographic surveillance of breast cancer survivors is situated at the crossroads of numerous different specialist areas of breast cancer control and management. The solutions for current problems probably lie in some important modifications in the conventional screening procedure that are underway or under study. These developments appear to be directed towards a partial modification of the screening rationale, with an adaptation to meet the diversified breast care needs of women. The complexity of the matter constitutes a call to action for several entities to eliminate the barriers to effective research in this field.

## Background

The prevalence of breast cancer (BC) in developed countries has steadily increased over recent decades due to improvements in detection and treatment of the disease. The immediate and long-term health needs of patients are receiving increasing attention. This is also the case for preventive care and screening services.

The subject of this article is the unresolved question of whether patients treated for BC should receive mammographic surveillance in the clinical or screening setting and, thus, whether they should be excluded from, or invited to, organised mammography screening programmes for the general female population. Up until recently this question was seldom raised [1,2], but with the increasing diffusion of screening, which has been and continues to be a major factor for the increase in the prevalence of BC, the situation has changed. The mammographic surveillance of BC survivors deserves careful and thorough consideration.

The many correlates of this problem should be placed in the wider context of cancer survivorship research. This area of translational research, which covers a broad range of topics including preventive measures and screening for patients with a history of cancer, is growing exponentially and is expected to translate into new evidence-based health care models and programmes. The development of rational approaches to continued breast surveillance is a key element of breast cancer survivorship agenda.

The problem of who should be responsible for the mammographic surveillance of BC survivors involves several specific points of interest. First, it is related to some emerging research subjects in the area of BC screening, such as the idea of risk-tailored screening. Secondly, and from a larger perspective, establishing whether women with a history of BC should be excluded from organised screening programmes is correlated with numerous aspects of BC control and management. Due to this multifaceted profile, the provision of mammographic surveillance for BC survivors raises wider questions concerning the incorporation of screening into primary care services and the interaction between public and personal health services.

The objective of this article is to discuss the many and contradictory aspects of this matter and the possible solutions. The basis for the discussion is an international overview of screening guidelines and practices for BC survivors. The work is directed at health policy makers, health managers, screening authorities, and primary care physicians. However, given the (problematic) interrelationship between mammographic surveillance and clinical follow-up for BC, it might also be of interest to oncologists and other hospital specialists. The article specifically addresses population-based, organised mammography screening programmes (with active call/recall service), as implemented in public, tax-financed health care systems. However, some of the information reported here comes from private insurance-based health care systems.

The points dealt with in the article are the following: the size of the problem, including the prevalence of BC and the risk of ipsilateral relapse and new contralateral cancer; the benefit of mammographic surveillance; the published guidelines for its frequency and duration; the strategies currently being used in organized screening programmes and in the clinical setting; the current levels of compliance with mammographic surveillance in either setting; considerations that may contribute to the rationale behind the decision-making on the provision of the service; the current challenges and potential developments that may be expected in the future; and the practical solutions that can be implemented to improve the situation in the short term.

## Discussion

### Size of the problem

#### Prevalence of BC

To calculate the complete prevalence of BC and to identify all prevalent patients, a long history of cancer registration and follow-up for vital status is needed. In the great majority of countries and regional areas with organised screening programmes, this requirement is not or only partially met. An alternative approach is to use statistical models such as those based on equations that relate mortality and prevalence to incidence and survival probabilities. Observed and estimated data provide a fairly consistent picture of current prevalence of BC among women living in the western world. In the United Kingdom [3], the Netherlands [4], Italy [5], the United States [6], and Australia [7], at different points in time between 2000 and 2009, the prevalence rate was approximately 2-2.5% around the age of 50 years and 4-6% around the age of 70 years. Over the past few decades, a steady increase has been observed. In the United States, for example, the rate has been projected to increase by 40% between 2005 and 2015.

#### Risk of ipsilateral relapse and new contralateral cancer

The incidence of ipsilateral relapse after breast-conserving surgery and radiotherapy is in the range of 0.5-1% per year [8-10]. This rate remains constant over time and, thus, the cumulative risk increases progressively, even after 15 years [9]. According to some observations [11], most ipsilateral relapses occur in the first 3 to 5 years. However, this is the case for true local recurrences alone, which follow the same decreasing time trend as distant metastases. The trend of new cancers in other areas of the treated breast is the opposite, as is that of new contralateral cancers. As a consequence, the pooled incidence of locoregional relapse in either breast remains constant for over 15 years at 1-1.5% per year [11,12]. By implication, the total cumulative risk increases with time, and the majority of relapses occur more than 3 years after treatment [12].

Among women aged above 50 years, a personal history of in situ or invasive BC conveys a risk of second contralateral invasive cancer of 1.5-1.75 relative to women with a negative history. This is equivalent to saying that the risk of second contralateral BC is greater than the familial risk of primary BC [13].

### Effectiveness of breast surveillance

#### General evidence of effectiveness

The evidence that mammographic surveillance for early detection of ipsilateral breast relapse or new contralateral cancer reduces the risk of death among BC survivors is less than one would expect. No randomized studies have been conducted, and observational studies have often been hampered by small sample size and low quality of design [14]. Their results have suggested that regular annual or biennial mammography is associated with a mortality reduction of varying magnitude [15,16] or a long-term survival benefit independent of lead time [17]. A recent review concluded that there is evidence of a potential benefit for asymptomatic second BCs compared with symptomatic ones in various surveillance strategies that include mammography [10].

The value of routine clinical examination in detecting a relapse in the ipsilateral breast or axilla or a new contralateral cancer is not certain. Patients with clinically detected ipsilateral breast relapse have a poorer survival than those whose relapse is detected by mammography or breast self-examination. Moreover, the proportion of relapses detected on routine clinical examination has decreased over time, from 45% according to reports published before 2000 to 15% according to more recent data. In other words, the contribution of mammography shows increasing importance because of technical improvements and quality assurance [18]. As a consequence, the question is not - or not just - whether clinical examination should complement mammography, but whether the image quality of mammograms is sufficiently high to permit the omission of clinical examination.

Breast self-examination continues to have a role in the follow-up of BC patients. The proportion of relapses detected by patients themselves is still about 35%. However, breast self-examination detects more ipsilateral relapses than contralateral relapses, which suggests a lack of patient awareness that the risk increase applies to both breasts [18].

There are no studies showing treatment or survival benefit from an earlier detection of asymptomatic relapse of BC using magnetic resonance imaging [19]. Similarly, there are insufficient data to support the routine use of breast ultrasound in post-treatment surveillance [20].

#### Evidence of effectiveness in different settings

Central to the present discussion is the lack of evidence that the effect of regular mammographic surveillance varies between the clinical and the screening setting. It has never been demonstrated that BC survivors cannot be screened successfully in an organised screening programme [1]. Moreover, there are no studies showing that the provision of mammography at intervals of less than 12 months is more effective than at 1- or 2-year intervals. It has been proposed that studies addressing this issue be based on organised screening programmes [10].

### International comparison of breast surveillance guidelines and practices

#### Surveillance guidelines

Due to the lack of evidence-based data on the most appropriate protocols for breast surveillance of BC patients, published practice guidelines give somewhat conflicting information. No consensus exists about how frequently patients should undergo mammography and clinical breast examination (if any), for how long they should be seen at short intervals, and whether or when it is appropriate for them to return to normal screening [2,10,21,22].

For example, the United Kingdom NHS National Institute for Health and Clinical Excellence guidelines of 2009 recommend that patients with early BC, including ductal in situ carcinoma, be offered annual mammography until they enter the national screening programme. Patients who are already eligible for screening should have annual mammography for 5 years. For patients who reach the screening age as well as those who complete 5 years of annual mammographic surveillance, it is recommended that the screening frequency be stratified according to the patient risk category [20].

The British Association of Surgical Oncology recommends that patients be followed-up for 5 years, although this period may vary with local protocols. As far as the frequency of mammography is concerned, the Association refers to the guidelines from the Royal College of Radiologists which suggest 1- to 2-year intervals for up to 10 years [23].

The 1998 clinical practice guidelines for the care of BC from the Canadian Steering Committee [24], as well as the 2005 guideline update [25], recommend that mammography and clinical breast examination be more frequent in the first few years and be performed indefinitely at approximately 1-year intervals, although the frequency may be adjusted according to individual patient's needs.

The 2006 update of the American Society of Clinical Oncology guidelines recommend that all patients have a careful history and physical examination every 3 to 6 months for the first 3 years, every 6 to 12 months for years 4 and 5, and annually thereafter, and that a mammogram be obtained at least 6 months after completion of radiation therapy and every year thereafter [26]. It is of note that no advice is given regarding patient discharge to primary care, whereas previous guidelines of 1999 suggested that this should occur after 10 years [12].

Finally, the European guidelines for mammography screening recommend that screen-detected BC patients have annual mammography [27] or periodical (not otherwise specified) physical examination and mammography [28]. In the section dedicated to annual surgical follow-up [29], it is stated that bilateral mammograms should be obtained and should have a technical quality equivalent to those provided in the organised screening programme. One could suggest that follow-up mammograms of exactly the same quality as screening mammograms can easily be found in screening centres. Unfortunately, the text does not address where follow-up mammography should be performed nor for how long. However, in the section that deals with the definition of the population eligible for screening [30], it reads that patients with previous BC (probably including both screen-detected and prevalent BC patients) *" ... may ..." *be excluded from the programme. In other words, and contrary to a widespread belief, the exclusion of BC survivors from organised screening programmes is presented as an option, not a necessity.

A Table in the Additional file 1 gives a summary of the above surveillance guidelines.

One key point should be noted here. Most guidelines place emphasis on providing frequent mammography and clinical examination in the first 3 to 5 years after diagnosis, and suggest that follow-up may be less frequent or even terminated thereafter [12,18]. This is not in accordance with the temporal pattern of the risk of relapse. The pooled incidence of true local recurrence, new BC in other areas of the ipsilateral breast, and new contralateral BC is constant for at least 15 years [11,12]. By implication, if any breast surveillance is to be offered, there is no obvious reason why it should be made less frequent or even discontinued after a few years [18]. Current guidelines seem to be based on the assumption that there exists an acute high-risk phase of follow-up. In fact, this is only the case for the detection of scar recurrences and distant metastases. It clearly appears that most current guidelines are influenced, if not dictated, by surgical and oncological priorities. By implication, they have limited or no relevance in terms of long-term surveillance for the early detection of new ispilateral and contralateral diseases.

This explains why most guidelines pay so much attention to the frequency of follow-up mammography and so little to the complementary question of which health setting is most appropriate. The option of providing mammographic surveillance through organised screening programmes is simply not taken into consideration.

#### Surveillance practices in organised screening programmes

The inconsistency of practice guidelines, their emphasis on the early post-treatment phase, and their ambiguity and omissions are among the major reasons why BC survivors are permanently or temporarily or partially excluded from many - but not all - organised screening programmes worldwide. It is important to note that there is also a lack of consistency and clarity in policies and procedures within the same countries.

In 1999, a survey of organised screening programmes and mammography registries in 25 member countries of the International Breast Cancer Screening Network was performed. The objective was to determine what specific data relating to screening monitoring were collected. Information on whether women attending screening had a history of BC was available from 15 countries. Most of the national representatives of these countries stated that women with a history of BC were specifically not invited to screening [31].

In Sweden, for example, women who ask to be excluded and women with previous BC are not invited, and this is also the case for women with newly diagnosed, screen-detected cancer [32]. In Poland [33] and Hungary [34], non-eligible women are defined as those who have undergone surgical treatment for BC. In Switzerland, gynaecologists and radiologists have agreed that BC survivors need closer medical follow-up outside organised screening programmes [35]. In Canada, the target population at the national level is defined as asymptomatic women aged 50-69 years with no prior diagnosis of BC [36]. Recently, some provincial programmes have started to include BC survivors. These women, however, are excluded from routine statistical reports for the first 5 years after diagnosis [37].

The Italian Group for Mammography Screening guidelines do not establish a management standard for women with a history of BC. However, they state that only patients who are known with certainty to be followed-up in the clinical setting can be excluded from an organised screening programme, and that the exclusion criteria should be clearly defined during the planning phase [38]. As the planning and implementation of screening are decentralized to the health care district level, approaches differ in many respects [39-41]. In general, BC survivors are indefinitely excluded. In situations where identifying patients is impossible or impractical, the invitation letter asks them to inform the screening service of their status, with exclusion occurring post-hoc. In the health care district of Rome, those patients who are being followed-up clinically are asked to send a signed declaration to the screening service in which they state their willingness to be excluded [42].

There are important organised programmes that use different, if not opposite, approaches. For example, it is not the policy of the United Kingdom NHS Breast Screening Programme to exclude BC survivors. The only women who are discontinued from the programme are those with bilateral mastectomy, those who are considered by general practitioners to be medically unsuitable due to terminal illness, and those who have explicitly asked not to be invited. In all other circumstances, women have the opportunity of making an informed choice about whether or not to accept a screening invitation on each and every occasion it is made [43]. The United Kingdom Society of Radiographers has suggested that patients with a recent history might be followed-up outside the programme [44].

In Denmark, all women aged between 50 and 69 years at the start of an invitation round are invited to screening unless they have specifically asked to be excluded [45].

In Australia, the national mammography screening programme selects women on the basis of age alone (50-69 years) because "...*The basis on which women are eligible for screening must be clear and unequivocal*". On the website of the BreastScreen Australia programme, it also reads that "*Generally there is no dispute about a women's age..*." and that "*Any policy is only effective if it is able to be implemented*" [46]. This is obvious, but so often forgotten.

The screening management of BC survivors is not a binary option, *i.e*. exclusion or inclusion. It must be borne in mind that organised screening programmes no longer have a static design. It may happen that BC survivors are systematically invited and managed with non-standard procedures. One of these is the so-called early or short-interval rescreen, an opportunity that may be extended to inadvertently invited BC survivors (when their status is unknown to the service). Early rescreen may be used for several other conditions including poor technical quality of mammograms, high levels of mammographic breast density, hormone replacement therapy use, and family history of BC [47-49]. Where and with what frequency early rescreen is practiced, however, is hard to establish. Information is sparse. In Australia, the reasons for offering screening more often than every 2 years include a previous diagnosis of in situ carcinoma [50]. In northern Italy, BC survivors inadvertently invited are among the 0.6% of total screening participants who are placed on early rescreen [49]. A survey of NHS mammography screening centres [47] showed that 1.1% of total screening participants underwent early rescreen and that 21% of these were made up of BC survivors. In Canada, the total estimated rate of early rescreen is above 10% [37].

Another hybrid model of BC survivor management, which combines screening with a clinical service, has been developed in the health care district of Rome. Apart from some exceptions, all women receive a personal invitation to participate in the organised screening programme. BC patients who are not followed-up elsewhere are asked to contact the screening centre to arrange a follow-up visit that is equivalent to an assessment for a positive mammography result, after which they are referred for specialist oncology consultation [42].

These are only a few examples of the solutions that have been adopted for the provision of mammographic surveillance of BC survivors. Given the fact that organised screening activities are highly decentralised in most countries, it can be assumed that the way in which BC survivors are managed varies to a far greater extent than is suggested by the information available.

#### Surveillance practices in the clinical setting

Variability in approaches to breast surveillance of BC survivors is not confined to organised screening, but is also observed in the clinical setting [20]. A survey of a random sample of symptomatic breast imaging units in England documented an impressive disparity of protocols [51]. The same patient, depending on her place of residence, can be discharged to the national screening programme immediately after treatment or have an annual mammography for 10 years, although most units recommend a 5-year duration of follow-up. In certain units, the schedule for patients treated with breast conserving surgery is based on a different frequency of mammography for the ipsilateral and contralateral breast. Such a scheme has a high risk of being misunderstood by patients, as suggested by the fact that they are more aware of the risk of ipsilateral relapse [18], and of being mismanaged by the follow-up service.

In England and elsewhere, the degree of within- and between-centre variation in follow-up strategies has probably increased over time because of a qualitative change in the service workload. In all populations targeted by screening, the growth in the number of BC patients to be followed-up is associated with a broadening range of clinical situations due to the fact that the presence of screen-detected cancers amplifies the heterogeneity of biological characteristics of the disease [52]. This, in turn, may widen the spectrum of proposed follow-up schedules. Some breast imaging units use one or more tumour-related risk factors for local recurrence to modulate the duration and frequency of examinations [51].

### Attendance to mammographic surveillance

Uncertainties about the optimal setting for mammographic surveillance, together with the variability of practices, may create confusion among BC patients invited to screening or referred to the clinical setting. In turn, this may adversely affect their compliance, especially in the medium-long term [2].

To the author's knowledge, there are no data on the proportion of BC survivors excluded from an organised screening programme who present for mammographic follow-up in the clinical setting. The only available studies on attendance of BC patients at clinical breast imaging units are from the United States, where approximately 80-85% patients with early BC return for mammography examination within 1 [53-55] or 2 years [56,57] after treatment. Attendance at annual mammographic follow-up, however, decreases with unexpected rapidity to approximately 65% in the fourth or fifth year [53,55]. Such a level is acceptable only for the general population of women at average risk of disease. Unfortunately, but in line with guidelines that recommend a decreasing intensity of follow-up over time, there are no data on the degree of attendance over longer time periods.

It is curious to note that one of the suggested explanations for mammography underuse among BC survivors is that their concern about cancer recurrence is greater in the first years after treatment [58]. Whilst this interpretation is probably correct, it must be acknowledged that the same concerns are apparently shared by the experts who developed the surveillance guidelines and by those who assessed their implementation.

Data are also lacking on the response rate of BC patients invited to organised screening at both short and standard interscreening intervals. A single, large study from the United Kingdom reviewed a cohort of BC survivors after 5 or more years of survival, *i.e*. after their return to the organised screening programme [59]. These women were 22% less likely to accept invitation than non-cancer controls. It is surprising that so little is known about mammography use by BC survivors in countries other than the United States [60].

A similar paucity of data extends to the health outcomes of these patients. The exclusion of BC survivors from organised screening programmes is based on the assumption that they will have an advantage from undergoing clinical mammography in the sense that a second BC would theoretically be detected at an earlier stage. In actual fact, the only available tumour stage data are from selected clinical series [17]. Population-based studies are precluded by the fact that general cancer registries report new ipsilateral and contralateral primaries, but not recurrent cancers.

### Arguments for and against different settings for mammographic surveillance

There are several other considerations that may contribute to the rationale behind the decision-making on the provision of mammographic surveillance for BC survivors.

#### Clinical control over mammography

Oncologists and other hospital specialists tend to retain direct control over the clinical investigations that are indicated to detect a cancer recurrence, and this is also the case for follow-up mammography. As intensive screening for asymptomatic metastatic relapse is not recommended because it does not improve survival or quality of life [10,61], the hospital follow-up of BC patients appears to be targeted at the following objectives: (1) to monitor ongoing adjuvant treatment and side-effects; (2) to review the patients who are participating in clinical trials; (3) to give information on general health, diet and exercise; (4) to identify and treat psychosocial problems; (5) to manage comorbidity; (6) to detect symptomatic true ipsilateral breast recurrences and axillary recurrences by clinical examination; and (7) to detect new asymptomatic cancers in both the treated and the contralateral breast using mammography [18,20,62].

Objectives #1 to 6 require a patient-physician interaction, which can take place in a hospital outpatient oncology or surgery clinic or, after the transfer of responsibility, in the primary care setting or elsewhere in the clinical health care environment [62]. This is not the case for the mammographic detection of a new BC. Oncologists, surgeons, and primary care physicians can only prescribe, but not perform, mammography. The only control that they can exercise over it is over its frequency. In other words, the only reason for which they may prefer that BC survivors be followed-up in a clinical radiology setting is that the frequency of mammography can be higher than in a screening setting. However, it is a fact that the provision of short-interval mammography for high-risk women is also relatively common in organized screening programmes [37,47-49].

#### Patients' views about mammographic surveillance

BC patients' views should be given closer attention when planning post-treatment follow-up. A systematic review of qualitative studies reporting on patients' views and preferences about follow-up for any type of cancer has shown that they attach importance to the regularity of follow-up appointments, expertise of specialists, rapid access to tests, and continuity of care and information [63]. There is no published information about BC survivors' attitudes towards the different options available for mammographic surveillance.

#### Limitations of cancer registration

The exclusion of women with BC from an organised screening programme involves the identification of all prevalent as well as incident BC patients. The latter should also be promptly notified to the screening service. This is impossible to achieve in several countries and regional areas due to the absence of cancer registration or limitations in geographic coverage or time coverage. Additional problems may be caused by the latency time of cancer registries, legal restrictions of access to their files, and poor quality of record linkage procedures.

Many mammographic surveillance protocols recommend that BC patients be returned to normal screening after some years (generally 5) of exclusion [51]. It is interesting to note that the latency time of cancer registries accounts for most, if not all, of this period. This means that during the latency time only screen-detected cancer patients are known to the screening services and can be excluded from invitation. The rest of incident cancer patients (a subgroup of the same order of magnitude) cannot be excluded until the latency time has expired. This confirms that current mammographic surveillance protocols are designed for the physician-patient setting, not for population-based screening programmes.

#### Inequalities in patient management

The health inequalities facing BC patients during post-treatment breast surveillance are not adequately perceived. The limitations of cancer registration translate into a mixed practice of both exclusions and inclusions from organised screening, *i.e*. different types of management being adopted by the same service for patients with the same condition. This is unacceptable in principle, may negatively affect the image of the screening programme, and may be associated with medico-legal hazards. BC patients excluded from organised screening programmes are subject to further inequalities when they present for mammographic follow-up in the clinical setting. As pointed out above, the current multiplicity of different recommendations results in an extreme diversity of protocols in use [51].

#### Rate of abnormal mammograms

It has been suggested that excluding BC survivors from organised screening programmes would be justified if their rate of abnormal mammograms requiring subsequent recall for diagnostic work-up was significantly greater than that of the general target population. If this was the case, the level of inconvenience for these women would be excessive and immediate evaluation in a diagnostic setting would appear more appropriate [1].

Theoretically, patients treated with conserving surgery and radiotherapy are expected to have an increased prevalence of mammographic abnormalities because they have an altered breast architecture which changes over time and causes difficulties in radiological interpretation [51]. To what extent this actually affects the recall rate for diagnostic assessment, however, cannot be established from the little data available [1,64]. At present, it is premature to recommend that BC survivors be followed-up in the clinical setting based only on the presumed increase in abnormal mammograms. Incidentally, it should be noted that this paucity of data extends to all performance measures of mammographic surveillance including cancer detection rate, positive predictive value, and interval cancer rate [10].

#### Biases in screening monitoring

Despite the lack of information, it should be taken into account that including BC survivors in the target population may influence the results of an organised screening programme. In particular, this may be the case for indicators related to the prevalence of mammographic breast abnormalities and of BC. The biasing effect of the presence of BC survivors could be considered, at least in part, a disadvantage from a quality monitoring point of view, *e.g*. in cross-national and international comparisons of screening results. This problem, however, can be overcome by excluding women with a personal history of BC from data reports and analyses. There is no "statistical" need to exclude them from screening. This approach, which has been proposed by the European guidelines for mammography screening [30], is currently being used in some provinces in Canada where BC survivors are invited to screening but withdrawn from data reporting for the first 5 years after their diagnosis [37].

#### Cost considerations

Economic evaluations have concentrated on comparing follow-up, including mammographic follow-up, of BC in primary care versus secondary or specialist care [62,65]. The provision of follow-up mammography in the screening setting is not considered an option worthy of cost-effectiveness and cost-minimisation analysis.

### Current challenges

Important changes are either underway, in preparation or at the research stage in procedures for BC screening and post-treatment management of women with BC. These developments have a potential to affect the way in which the problems with mammographic surveillance of BC survivors are perceived and treated.

#### BC survivorship research

Preventive health care needs of BC survivors are a specific topic of BC survivorship research. The term cancer survivorship encompasses the many physical, psychosocial, and economic sequelae of cancer diagnosis and treatment as well as the related short- and long-term needs of the patients. Cancer survivorship research aims at developing an evidence base for optimal follow-up care practices using a multidisciplinary approach.

The most important objective remains the achievement of a higher level of evidence of effectiveness of mammographic surveillance of BC survivors in reducing mortality. Other major issues to be addressed include the effectiveness of surveillance as specifically provided in the screening setting [10] and the relative effectiveness associated with different time intervals. It is clear that studies aimed at evaluating these potential benefits will be impossible to conduct in countries and regional areas where BC survivors are not eligible for organised screening programmes.

Obtaining access to appropriate care services through delivery systems that are complementary and not duplicative [66] is a primary area of improvement. From this point of view, the establishment of coordination schemes between specialists and primary care physicians, which includes formalization of the transfer of care [53,67,68], is considered insufficient to substantially improve patient management [66]. The coordination strategy should extend to a larger number of health services. Numerous models for delivering survivorship care have been proposed [63,69]. The complicated question of how mammographic surveillance could be incorporated into these comprehensive care planning processes is among the areas of BC survivorship where more research is needed.

A patient's family members are considered part of the survivorship experience. Indeed, post-treatment care is increasingly viewed as a spectrum of interventions aimed at improving the health and lives of cancer survivors and their families [66]. On the one hand, the well-being of the millions of family members of cancer survivors, many of whom are themselves at high risk for cancer, is a matter of increasing concern. On the other hand, it is now recognised that household members have a role in survivors' health-related outcomes. From this perspective, there is an objective interrelationship between the management of the personal high-risk condition of a BC patient and the management of the high-risk condition that affects her female family members. It might be worth studying whether there could be some benefit from coordinating these two parallel surveillance activities.

#### Socio-economic factors of adherence to mammographic surveillance

The expansion of BC survivorship research will help us to understand and control the effects of socio-economic factors on patient attendance at post-treatment mammographic surveillance. The observed underuse of mammography is mostly related to clinical and medical factors such as advanced patient age, comorbidities, late disease stage of primary cancer, insufficient teamwork between oncology specialists and primary care physicians [67,68], and inadequate instructions on preventive care. Studies on the underuse of screening mammography in the general population and on follow-up failures after a positive screening result indicate the need to investigate the role of socio-economic factors in explaining the degree of patients' adherence to recommendations.

#### Risk-based surveillance protocols

The feasibility, cost-effectiveness, and potential benefits of different risk-based protocols for mammographic follow-up in the clinical setting are important areas of clinical research. Efficient protocols could facilitate the discharge of low-risk patients to the organised screening activity. Many tumour-related risk factors for local recurrence have been identified which could be used to modulate the duration and frequency of mammographic follow-up. A small number of breast imaging units are already using one or more of these factors to schedule appointments [51].

Further research in the form of a controlled trial is necessary to determine optimal surveillance regimens. In this respect, however, a problem exists in that obtaining ethical approval may be difficult. Although there is no evidence to support current guideline recommendations for annual mammography, it could be deemed unethical to randomly assign patients to receive a lower level of surveillance [51].

#### Risk-tailored screening

Inviting BC survivors to participate in an organised screening programme inherently conveys the innovative and controversial concept of tailoring the screening procedure to the personal risk of disease. This would involve the implementation of risk assessment in screening facilities [70] and major changes in service delivery arrangements. Risk-tailored screening would provide an opportunity to both increase the impact on mortality and reduce the human and economic cost of screening [71]. For example, the frequency of screening in women who are assessed as being at lower risk levels could be reduced. At present, however, we still need to learn how to combine the many known risk factors for BC [71,72]. Moreover, risk-tailored screening will need to be evaluated in prospective trials, although it has been proposed that surrogate indicators of effectiveness (such as tumour stage at diagnosis) be used [71].

Such a perspective warrants a research effort. In a parallel line of investigation, it has been hypothesised that quantifying both the individual risk of multiple BC and the familial risk, as well as their interactions, in populations exposed for extended periods to organised screening programmes could be helpful to identify optimal preventive strategies [13,73].

#### Screening after age 69 years

In the Netherlands women are routinely invited to organised screening programmes up to 75 years of age, in France and Sweden up to 74 years. The extension to 73 and 74 years is being planned in the United Kingdom and some Italian administrative regions, respectively. Recommendations on how long to continue screening mammography on a spontaneous basis are also expected to evolve.

Raising the upper age limit for organised screening may influence the mammographic surveillance of BC survivors in two ways. First, assuming that they are not invited, it is reasonable to expect that they will be followed-up in the clinical setting at least until the age of 74. A survey of breast imaging units in England has shown that variability in the duration of mammographic follow-up is especially pronounced for patients aged 70 years or older, with a range of 0 to 15 years [51]. The cessation at 69 years probably reflects the most common upper age limit for organised screening, although the effectiveness of mammography for women aged up to 74 years has been demonstrated in several randomized trials.

Second, the inclusion of women aged 70 years or older in organised screening programmes may facilitate the inclusion of BC survivors themselves. An Australian modelling study aimed at estimating the costs and benefits of continuing screening mammography after 69 years of age concluded that a benefit is more likely in women who are at high risk of death from BC and those at low risk of death from other causes [74]. In the event the upper age limit for screening is raised, BC survivors should be considered candidates for invitation, provided that primary care physicians select those who are eligible on the basis of evaluation of life expectancy and severity of comorbidities.

Consultation with primary care physicians is important for all elderly women, with or without a personal history of BC. Both populations need to be provided with guidance on mammography, regardless of whether they will be screened in the clinical setting or in an organised screening programme. In the latter case, however, a subtle change in the design of screening inevitably occurs. As the participation of elderly women is dependent on an assessment of life expectancy performed by primary care physicians, it can be said that they are screened for eligibility before undergoing screening for BC. This two-stage procedure is equivalent to a selective screening for women who have low competing risks of death and a potential for benefit from mammography.

#### Breast units

Medical organisations are increasingly recommending that breast diseases be cared for by specialists working as a team in multidisciplinary breast units. The section of the European guidelines for mammography screening which defines the ideal requirements for breast units introduces a new concept of the interrelationship between population-based screening activities and clinical breast care services [75]. It is stated that organised screening programmes should be based within a breast unit, and that any radiologist who reads clinical mammograms should participate in the organised screening programme in those countries in which this is established.

This model of provision of breast care, in which the separation of screening from clinical breast services is no longer straightforward, may also have implications for breast surveillance of BC survivors. European guidelines recommend that such patients be followed-up in a breast unit under the direct supervision of one of the surgeons, and that any necessary imaging be carried out at the same visit. In line with these criteria, but also with the aim of optimising the use of resources, it could be proposed that patients be followed-up in the clinical setting of the breast unit but using the personal call/recall service of the organised screening programme. This would improve attendance rates and would facilitate the subsequent return to the programme.

The process of the establishment of breast units in Europe is in its initial stages, and their organisation varies greatly between countries [76]. It is possible that a variety of models of cooperation between screening and clinical services will be proposed in the future.

#### BC registration

Where there is a policy to exclude BC survivors from organised screening programmes, such individuals need to be systematically identified. In turn, this makes it necessary to improve the spatial and temporal coverage of cancer registration.

The introduction of a screening programme provides the motivation for expanding a general cancer registry or for implementing a special, screening-oriented BC registry, although optimal results are impossible [49]. Hospital discharge records, medical insurance claim files, treatment records, pathology report files, and notifications from primary care physicians are potential alternatives to cancer registry data when these are not (or not readily) available. However, data from hospital discharge records and medical insurance claim files are often very complex and require training and support. The frequent lack of a unique universal identifier causes additional difficulties because the quality of electronic record linkage procedures is always less than perfect. Rapid case ascertainment procedures can shorten, but not eliminate, the latency time.

### Possible interim solutions

Ongoing and potential developments in the area of BC cancer screening and care might offer solutions to problems arising from current patterns of breast surveillance of BC survivors. However, this is not expected to happen in the near future. In the interim, there are some practical corrective measures that could be implemented.

#### Primary care physicians

Many studies on the provision of mammographic surveillance have concluded that the coordination between the services involved needs to be improved. To this end, the first step is to better define the role and responsibilities of primary care physicians [67,68]. In health care systems where these are clear, such as in the United Kingdom, cancer survivors receive preventive care at similar levels to the non-cancer population. Where the role of primary care physicians is less clearly defined, patients receiving the best care are those who maintain contact with surgeons and oncologists after discontinuation of hospital follow-up [59]. Data from the United Kingdom have confirmed that primary care physicians order follow-up mammograms more frequently than hospital specialists [65]. Studies from the United States, conversely, have led to the hypothesis that mammography rates decrease rapidly after treatment because primary care physicians have insufficient knowledge of the benefit of surveillance [53].

#### Local flow charts

Another option to improve the coordination of care for BC survivors, including breast surveillance, would be to introduce practice guidelines and, more specifically, flow charts on a local basis. These would be designed to assist all health care providers in the implementation of follow-up, would illustrate the crucial points in patient management, and would eliminate redundant or inappropriate actions.

The implementation of a population-based screening programme, which increases the number of BC survivors and creates new inequalities, offers a unique opportunity for local public health authorities to promote the development and adoption of flow charts for mammographic surveillance. As research evidence of the effectiveness of different protocols is lacking, flow charts should be based on empirical experience. A realistic consensus between practicing physicians at the community level, with the identification of distinct roles for the screening service and clinical practitioners, would be much easier to achieve than a large-scale consensus between experts. Moreover, a local approach would offer the advantage of greater flexibility in the application of health regulations from higher levels of government.

#### Screen-detected patients

In a population targeted by an organised screening programme, screen-detected patients account for approximately half of BC incidence. By definition, they are known to screening centres and can immediately be excluded from subsequent screening rounds. It seems more reasonable, however, for the mammographic follow-up of this subset of BC patients to take place in the same setting in which they were diagnosed. For example, accreditation standards recently introduced in an administrative region of northern Italy stipulate (1) that all organised screening centres should offer annual follow-up mammography to patients with screen-detected BC, (2) that these should receive personal ad-hoc invitation letters, and (3) that their mammographic follow-up status should be checked at regular intervals by the same staff who check whether women with a positive basic screening result have undergone diagnostic assessment [77].

It is doubtful whether this policy could be implemented, in its entirety, in other health systems and under different health regulations, especially with respect to the status and role of primary care physicians. However, this model illustrates the growing idea that breast cancer screening should comprise multiple surveillance procedures aimed at subsets of the population with different risk levels.

#### Recording of screening data

The proportion of BC patients who participate in organised screening programmes, either because of incomplete exclusion or because they are invited by design, is probably much greater than is commonly assumed. However, there are no reliable data to confirm this. The status of cancer survivors is seldom recorded in mammography databases, and there may also be some confusion as to when to enter these women as clinical cases and when to include them again in the basic screening population. The return to basic screening has been called "a grey area" [47], which highlights the need for guidelines for the management and classification of patients.

In Europe, there is increasing awareness that the functions of the computer systems used in screening activities need to be improved. This also applies to completeness and consistency of the recorded items [78]. Until the hypothesis of risk-tailored screening has been prospectively studied with ad-hoc designs, it would be useful to have a code indicative of patient status to be entered in service databases. This would enable us to evaluate the attendance rate, the rate of abnormal mammograms, and the detection rate of second breast cancer among BC survivors [17].

#### A collaboration scheme between primary care physicians and organised screening programmes

Until ongoing and expected modifications in BC cancer screening and care provide substantial solutions to problems with breast surveillance of BC survivors, it is the author's opinion that many shortcomings could at least be mitigated by the adoption of an organisational model in which: (1) the primary care physician retains (or regains) full accountability for the follow-up care of BC patient; (2) the whole target population is invited to the organised screening programme; (3) the primary care physician advises the BC patient to decline the invitation to screening in the event of a recent or imminent clinical mammography; (4) otherwise, and after assessment of life expectancy and competing risks, he encourages the patient to participate; (5) after her first screening mammography, the patient is re-invited annually until the standard age of cessation; and (6) the mammography report is forwarded to the primary care physician, who supervises follow-up.

This approach has analogies with that being used in the United Kingdom NHS Breast Screening Programme. Its rationale is that: (1) it leaves unchanged the existing information flows to cancer registries; (2) it avoids inequalities in the public provision of mammography service; (3) it is in keeping with the view that patients who are not known with certainty to be followed-up in the clinical setting cannot be excluded from an organised screening programme; (4) it extends the provision of a fail-safe mechanism against discontinuation of mammographic surveillance; and (5) it decreases the likelihood of over-investigation.

## Summary

BC survivors are still a small but no longer negligible minority of women in the age range targeted by organised mammography screening programmes. Due to their increasing numbers, the problem of determining the most appropriate setting in which to carry out their mammographic surveillance can no longer be ignored. This article, that is based on an international comparison of policies and practices for such women, documents a poor level of evidence-based information, an absolute lack of consistency and clarity, and several points of irrationality. Breast surveillance of BC survivors, like other needs of this growing population, has not been addressed in a satisfactory manner and must be given a higher rank in the cancer survivorship research agenda.

The main aspects of the problem include: weak evidence that mammographic surveillance decreases mortality; a lack of objective evidence in favour of one setting or the other; a lack of evidence-based guidelines for the frequency and duration of surveillance; a disproportionate emphasis being placed on the first few years after treatment, probably dictated by surgical and oncological priorities; a variety of screening policies, as these women are permanently, temporarily or partially excluded from many - but not all - organised screening programmes worldwide; an even greater disparity of follow-up protocols being used in the clinical setting; a paucity of data on compliance to mammographic surveillance in both settings; and difficulty in coordinating the roles of health care providers. The problem of where and how to provide mammographic surveillance for BC survivors is situated at the crossroads of numerous different specialist areas of BC control. A solution will be found only by expanding the conventional theoretical boundaries of secondary prevention of BC.

Following this line of reasoning, it is probable that novel approaches to coping with mammographic surveillance for BC survivors will emerge from some important modifications in the conventional screening procedure that are underway, such as the early rescreen practice and the implementation of breast units, or are still undergoing research, such as the hypothesis of risk-tailored screening. Taken as a whole, these developments are directed towards a partial integration, at the functional or structural level, between BC screening and clinical breast services. This would necessarily involve modifications of the traditional screening rationale, with an adaptation to meet the diversified breast care needs of women.

Such a complexity constitutes a call to action for health policy-makers, screening authorities and specialists to eliminate the barriers to effective research into innovative models for breast surveillance of BC survivors. Until these models are developed, there are technical solutions in patient flow and care coordination that can alleviate the most serious shortcomings. A collective commitment to a comprehensive approach to these problems is needed.

## Abbreviations

BC: breast cancer; NHS: National Health Service.

## Competing interests

The author declares that he has no competing interests.

## Pre-publication history

The pre-publication history for this paper can be accessed here:

http://www.biomedcentral.com/1472-6963/11/249/prepub

## Supplementary Material

Additional file 1 **Summary of selected breast surveillance guidelines for breast cancer survivors**. A table provides a summary of the type, frequency, and duration of breast surveillance for breast cancer patients according to practice guidelines from selected organisations. Click here for file
