# Supplementary material for: Should breast cancer survivors be excluded from, or invited to, organised mammography screening programmes?
Source: BMC Health Serv Res. 2011 Oct 4;11:249. doi: 10.1186/1472-6963-11-249 (PMC3203044; doi:10.1186/1472-6963-11-249)
Supplement: Additional file 1 — Summary of selected breast surveillance guidelines for breast cancer survivors. A table provides a summary of the type, frequency, and duration of breast surveillance for breast cancer patients according to practice guidelines from selected organisations. [file 1472-6963-11-249-S1.DOC]

**Table Summary of selected breast surveillance guidelines for breast cancer survivors**

| **Organisation (country)** | | **Year**  **[reference]** | | **Schedule** | | **Notes** |
| --- | --- | --- | --- | --- | --- | --- |
| NHS National Institute for Health and Clinical Excellence (United Kingdom) |  | 2009 [20] |  | - Annual mammography until the age of entry into the NHS Breast Screening Programme - For patients already eligible for the NHS Breast Screening Programme, annual mammography for 5 years |  | For patients who reach the screening age and those who complete 5 years of annual mammographic surveillance, the screening frequency should be stratified according to the individual risk category |
| British Association of Surgical Oncology (United Kingdom) |  | 2005 [23] |  | Not established |  | Reference is made to the Royal College of Radiologists’ guidelines (see below) |
| Royal College of Radiologists (United Kingdom) |  | 2003 [23] |  | Routine mammography every 1–2 years for up to 10 years |  |  |
| The Steering Committee on Clinical Practice Guidelines for the Care and Treatment of Breast Cancer (Canada) |  | 1998, 2005 [24,25] |  | Patient history, mammography and clinical breast examination every year and indefinitely |  | The frequency of mammography and clinical breast examination   - can be higher in the first few years - can be adjusted according to individual patient’s needs |
| American Society of Clinical Oncology (United States) |  | 2006 [26] |  | - Patient history and physical examination - every 3 to 6 months for the first 3 years - every 6 to 12 months for years 4 and 5 - annually thereafter - Mammography ≥6 months after radiation therapy and every year thereafter |  |  |
| European Breast Cancer Network *(European guidelines for quality assurance in breast cancer screening and diagnosis)*  (European Union) |  | 2006 [27,28] |  | Annual mammography or periodical (not otherwise specified) physical examination and mammography |  | The recommendation refers to screen-detected breast cancer patients alone |
